# Four new spider species of the *Pholcus phungiformes* species group (Araneae, Pholcidae) from South Korea

**DOI:** 10.3897/zookeys.1283.184235

**Published:** 2026-06-30

**Authors:** Chang Moon Jang, Jung Sun Yoo, Sue Yeon Lee, Seung Tae Kim

**Affiliations:** 1 Diversity Conservation Research Department, Nakdonggang National Institute of Biological Resources, Ministry of Climate, Energy and Environment, Sangju 37242, Republic of Korea Life and Environment Research Institute, Konkuk University Seoul Republic of Korea; 2 Wildlife Quarantine Center, National Institute of Wildlife Disease Control and Prevention, Ministry of Environment, Incheon 22382, Republic of Korea Diversity Conservation Research Department, Nakdonggang National Institute of Biological Resources, Ministry of Climate, Energy and Environment Sangju Republic of Korea; 3 R & D Center, Cellcuratio Co., Ltd., Daejeon 34054, Republic of Korea Wildlife Quarantine Center, National Institute of Wildlife Disease Control and Prevention, Ministry of Environment Incheon Republic of Korea; 4 Life and Environment Research Institute, Konkuk University, Seoul 05029, Republic of Korea R & D Center, Cellcuratio Co., Ltd. Daejeon Republic of Korea

**Keywords:** Diagnosis, mixed forest, morphology, new species, rocky area, taxonomy

## Abstract

Four new spider species of the genus *Pholcus* Walckenaer, 1805, *Pholcus baegamsan***sp. nov**., *Pholcus hongcheon***sp. nov**., *Pholcus namhae***sp. nov**., and *Pholcus yeongcheon***sp. nov**. belonging to the *P. phungiformes* species group in the family Pholcidae C. L. Koch, 1850, are newly described from South Korea. The new species presented here were hand-collected from rocky areas, such as rock walls and beneath rocks, in mountainous mixed forests.

## Introduction

**Figure 1.**
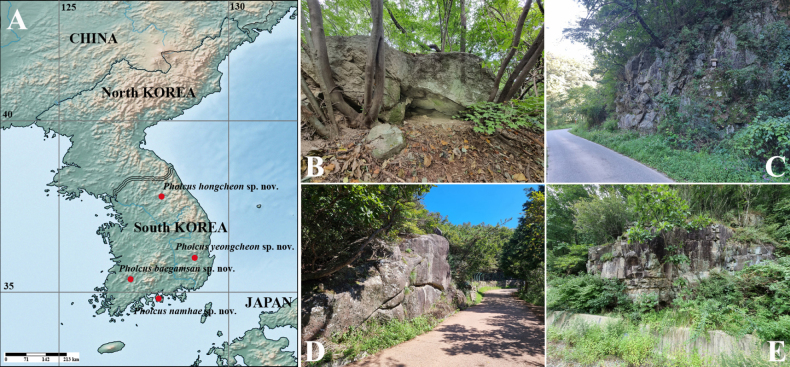
Distribution records and habitats of the new species. **A**. Distribution map; **B**. Mt. Baegamsan, Sunchang-gun (type locality of *P. baegamsan* sp. nov.); **C**. Mt. Garisan, Hongcheon-gun (type locality of *P. hongcheon* sp. nov.); **D**. Boriam Temple, Namhae-gun (type locality of *P. namhae* sp. nov.); **E**. Mt. Hwasan, Yeongcheon-si (type locality of *P. yeongcheon* sp. nov.).

Pholcidae C. L. Koch, 1850 is one of the most diverse and species-rich families within the order Araneae Clerck, 1757 comprising 2,060 species in 97 genera (World Spider Catalog 2026). The most diverse genus in the family, *Pholcus* Walckenaer, 1805 currently includes 434 species belonging to 21 species groups (Huber 2011; Huber et al. 2018; Yao et al. 2021; World Spider Catalog 2026). One of species group in the genus, *Pholcus phungiformes* species group is largely restricted to northern and northeastern China, the Korean Peninsula, and the Russian Far East and are mainly found in dusky, humid spaces like rock walls, road drains, and cave entrances in mountainous and coastal mixed forests (Kim and Lee 2004; Huber 2011; Yao et al. 2021; Seo 2014, 2018; Lee et al. 2021a, 2021b; Zhao et al. 2023; Lee et al. 2024; Jang 2025a, 2025b, 2025c; Lee et al. 2025). Recently, taxonomic studies on the *phungiformes* group have been actively conducted in Korea, with 61 species currently recorded (Paik 1978a, 1978b; Namkung and Kim 1990; Kim and Lee 2004; Seo 2004, 2014, 2018; Kim and Yoo 2008; Kim and Park 2009; Huber 2011; Kim and Ye 2014, 2015; Kim and Kim 2016; Lee et al. 2021a, 2021b, 2024, 2025; Jang et al. 2023, 2025a, 2025b, 2025c). Most mountainous temperate mixed forests in South Korea, consisting of both deciduous broadleaf and coniferous tree, contain numerous rocky areas suitable as habitats for spiders of the *P. phungiformes* species group, which contribute substantially to the composition and maintenance of biodiversity in mountainous ecosystems. During a survey of spider fauna in mountainous mixed forests from 2022 to 2023, four new spiders belonging to the *P. phungiformes* species group, *Pholcus baegamsan* sp. nov., *Pholcus hongcheon* sp. nov., *Pholcus namhae* sp. nov., and *Pholcus yeongcheon* sp. nov., were found inhabiting rocky areas (Fig. 1). The present study includes descriptions of these new species, a diagnostic key to the South Korean *Pholcus phungiformes* species group, distribution maps, and taxonomic photographs.

## Materials and methods

All specimens were collected by hands and preserved in 98% ethyl alcohol and external morphology was examined under a Leica S8APO (Singapore) stereomicroscope. Images were captured with a Dhyana 400DC zoom digital camera (China) mounted on a Leica S8APO and assembled using Helicon Focus 8.2.0 image stacking software (Khmelik et al. 2006). Measurements of body parts were made with an ocular micrometer and are recorded in millimeters. Internal genitalia of females were removed and treated in 10% KOH for 2 h before illustration. Leg measurements are shown as: total length (femur, patella, tibia, metatarsus, tarsus). Morphological terminology follows Huber (2011). Distribution map was produced by modifying SimpleMappr (Shorthouse 2010). All type specimens studied are deposited in the National Institute of Biological Resources (**NIBR**), Incheon and Konkuk University (**KKU**), Seoul, South Korea. The following abbreviations are used in the descriptions: **ALE** = anterior lateral eye, **AME** = anterior median eye, **PLE** = posterior lateral eye; **PME** = posterior median eye, **ALE-AME** = distance between ALE-AME, **ALE-PLE** = distance between ALE-PLE, **AME-AME** = distance between AMEs, **AME-PME** = distance between AME–PME, **PLE-PME** = distance between PLE–PME, **PME-PME** = distance between PMEs in the eye region; **L/d** = length/diameter in the leg measurement.

## Taxonomic account

### Family Pholcidae C. L. Koch, 1850

**Subfamily Pholcinae C.L. Koch, 1850**

#### Genus Pholcus

Taxon classificationAnimaliaAraneaePholcidae

Walckenaer, 1805

DE4BE1B2-D673-5277-8F43-3CBCDE6801D4

##### Type species.

*Aranea phalangioides* Fuesslin, 1775.

### *Pholcus phungiformes* species group

#### Key to the South Korean *Pholcus phungiformes* species group

**Table:** 

1	Bulbal process with pseudo-appendix	**2**
–	Bulbal process without pseudo-appendix	**38**
2	Bulbal process with two pseudo-appendices; epigynum subcircular, anterior plate straight posteriorly, anterior arch straight, pore plates semicircular, separated	***P. deokjeok***
–	Bulbal process with a single pseudo-appendix	**3**
3	Pseudo-appendix moderately long	**4**
–	Pseudo-appendix short	**29**
4	Pseudo-appendix with pointed tip	**5**
–	Pseudo-appendix without pointed tip	**15**
5	Pseudo-appendix curved	**6**
–	Pseudo-appendix not curved	**17**
6	Pseudo-appendix strongly curved	**7**
–	Pseudo-appendix slightly curved	**9**
7	Trochanter apophysis finger-shaped, with hair; epigynum subcircular, anterior plate straight posteriorly, anterior arch sinuous, pore plates elongate obovate, separated	***P. ungyo***
–	Trochanter apophysis finger-shaped without hair	**8**
8	Embolus longer than uncus, uncus subquadrate; epigynum subcircular, anterior plate curved posteriorly, anterior arch recurved, pore plates obovate, separated	***P. cheongogensis***
–	Embolus slightly longer than uncus, uncus auriculate; epigynum subcircular, anterior plate straight posteriorly, anterior arch slightly recurved, pore plates ovoid, separated	***P. gimsatgat***
9	Trochanter apophysis long and capitate, embolus straight; epigynum trapezoidal, anterior plate straight posteriorly and bulged posterolaterally, anterior arch strongly recurved, pore plates oval, contiguous	***P. kwanaksanensis***
–	Trochanter apophysis short and blunt, embolus slightly curved	**10**
10	Uncus rectangular; epigynum subcircular, anterior plate slightly curved posteriorly, anterior arch tectiform, pore plates semicircular and separated	***P. osaek***
–	Uncus auriculate	**11**
11	Uncus distally pointed, dorso-distal apophysis of procursus pointed	**12**
–	Uncus distally blunt, dorso-distal apophysis of procursus quadrate or claw-shaped	**13**
12	Procursus with angular ventrodistal apophysis, without retrolatero-distal apophysis; epigynum subrhomboidal, anterior plate curved posteriorly and bulged posterolaterally, anterior arch sinuous, pore plates triangular, separated	***P. montanus***
–	Procursus without ventrodistal apophysis, with claw-shaped retrolatero-distal apophysis; epigynum subpentagonal, anterior plate slightly curved posteriorly, anterior arch straight, pore plates oval, separated	***P. chiakensis***
13	Procursus with membranous ventral apophysis; epigynum subcircular, anterior plate curved posteriorly and bulged posterolaterally, anterior arch slightly sinuous, pore plates oblong, separated	***P. hwangjeong***
–	Procursus without ventral apophysis	**14**
14	Procursus with quadrate, pointed dorso-distal apophysis and membranous, triangular prolatero-distal apophysis; epigynum subcircular, anterior plate slightly curved posteriorly, anterior arch straight, pore plates rounded, separated	***P. pyeongchangensis***
–	Procursus with claw-shaped dorso-distal apophysis and membranous, serrated prolatero-distal apophysis; epigynum subcircular, anterior plate slightly curved posteriorly, anterior arch sinuous, pore plates oval, separated	***P. palgongensis***
15	Pseudo-appendix with rounded tip, trochanter apophysis triangular; epigynum elongate oval, anterior plate emarginate posteriorly, anterior arch sinuous, pore plates elongate oblong, separated	***P. chuncheonensis***
–	Pseudo-appendix with blunt tip, trochanter apophysis finger-shaped	**16**
16	Uncus horse-head-shaped, blunt proximally; epigynum elongate oval, anterior plate emarginate posteriorly, anterior arch sinuous, pore plates elongate oblong, separated	***P. yeongwol***
–	Uncus auriculate, pointed distally; epigynum subcircular, anterior plate slightly curved posteriorly, anterior arch emarginate, pore plates rounded, separated	***P. pajuensis***
17	Pseudo-appendix hooked	**18**
–	Pseudo-appendix blade-shaped	**25**
18	Trochanter apophysis long and rod-shaped, uncus subflabellate; epigynum subcircular, anterior plate straight posteriorly, anterior arch emarginate, pore plates triangular, separated	***P. seorakensis***
–	Trochanter apophysis short, uncus not subflabellate	**19**
19	Pseudo-appendix thumb-shaped; epigynum subcircular, anterior plate strongly emarginate posteriorly, anterior arch sinuous, pore plates oval, separated	***P. yeongcheon* sp. nov**.
–	Pseudo-appendix finger-shaped	**20**
20	Trochanter apophysis with hair; epigynum subcircular, anterior plate strongly curved posteriorly, anterior arch sinuous, pore plates obovate, separated	***P. socheunensis***
–	Trochanter apophysis without hair	**21**
21	Embolus strongly curved; epigynum subcircular, anterior plate strongly curved posteriorly, anterior arch slightly recurved, pore plates ovoid, separated	***P. uiseongensis***
–	Embolus slightly curved	**22**
22	Uncus subquadrate	**23**
–	Uncus auriculate	**24**
23	Uncus distally pointed, procursus with membranous and pointed ventro-distal apophysis; epigynum subcircular, anterior plate straight posteriorly, anterior arch sinuous, pore plates triangular, separated	***P. hwaam***
–	Uncus triangular distally, procursus with claw-shaped ventro-distal apophysis; epigynum subpentagonal, anterior plate slightly curved posteriorly, anterior arch short and strongly recurved, pore plates rectangular, separated	***P. yangpyeong***
24	Uncus distally pointed, procursus with curved and pointed dorso-distal apophysis; epigynum subcircular, anterior plate curved posteriorly, anterior arch strongly recurved, pore plates oblong, separated	***P. mino***
–	Uncus distally strongly serrated, procursus with claw-shaped dorso-distal apophysis; epigynum subcircular, anterior plate strongly curved posteriorly, anterior arch straight, pore plates oval, separated	***P. uksuensis***
25	Pseudo-appendix lanceolate, trochanter apophysis rod-shaped with apically curled tip; epigynum subcircular, anterior plate strongly curved posteriorly, anterior arch straight, pore plates oval, separated	***P. incheonensis***
–	Pseudo-appendix falcate or cultrate, trochanter apophysis finger-shaped with blunt tip	**26**
26	Pseudo-appendix falcate	**27**
–	Pseudo-appendix cultrate	**28**
27	Trochanter apophysis with hair, uncus auriculate; epigynum subcircular, anterior plate straight posteriorly, anterior arch emarginate, pore plates ovoid, separated	***P. chugok***
–	Trochanter apophysis without hair, uncus subquadrate; epigynum subcircular, anterior plate slightly curved posteriorly, anterior arch emarginate, pore plates elongate oval, separated	***P. hongcheon* sp. nov**.
28	Trochanter apophysis with hair, uncus pointed distally, procursus with pointed dorso-distal apophysis; epigynum subcircular, anterior plate slightly curved posteriorly, anterior arch sinuous, pore plates oval, separated	***P. deunggolensis***
–	Trochanter apophysis without hair, uncus curled distally, procursus with claw-shaped dorso-distal apophysis; epigynum subcircular, anterior plate strongly curved posteriorly, anterior arch sinuous, pore plates oval, separated	***P. okgye***
29	Pseudo-appendix pointed distally	**30**
–	Pseudo-appendix blunt distally	**36**
30	Pseudo-appendix triangular, uncus auriculate	**31**
–	Pseudo-appendix slightly curved or T-shaped, uncus elongate auriculate or triangular	**35**
31	Trochanter apophysis rod-shaped	**32**
–	Trochanter apophysis finger-shaped	**33**
32	Trochanter apophysis with blunt tip, procursus with membranous and pointed dorso-distal apophysis; epigynum subcircular, anterior plate straight posteriorly, anterior arch strongly recurved, pore plates ovoid, separated	***P. jindongensis***
–	Trochanter apophysis with curled tip, procursus with finger-shaped dorso-distal apophysis; epigynum urn-shaped, anterior plate straight posteriorly, anterior arch strongly recurved, pore plates oblong, separated	***P. seoulensis***
33	Trochanter apophysis with hair, procursus without prolatero-distal apophysis; epigynum subcircular, anterior plate straight posteriorly, anterior arch emarginate, pore plates obovate, separated	***P. noeun***
–	Trochanter apophysis without hair, procursus with prolatero-distal apophysis	**34**
34	Uncus with triangular indentation, procursus with curved and pointed prolatero-distal apophysis; epigynum subcircular, anterior plate sinuous posteriorly, anterior arch strongly recurved, pore plates oval, separated	***P. maepo***
–	Uncus with quadrate projection, procursus with membranous and pointed prolatero-distal apophysis; epigynum subcircular, anterior plate strongly curved posteriorly, anterior arch slightly recurved, pore plates oblong, separated	***P. simbok***
35	Pseudo-appendix slightly curved, trochanter apophysis spatulate, uncus elongate auriculate, embolus strongly curved; epigynum subcircular, anterior plate strongly emarginate posteriorly, anterior arch recurved, pore plates elongate oval, separated	***P. gajiensis***
–	Pseudo-appendix T-shaped, trochanter apophysis finger-shaped, uncus triangular, embolus slightly curved; epigynum subcircular, anterior plate slightly curved posteriorly, anterior arch emarginate, pore plates oval, separated	***P. woongil***
36	Trochanter apophysis short and thumb-shaped, procursus without dorsal apophysis; epigynum subcircular, anterior plate emarginate posteriorly, anterior arch recurved, pore plates elongate oblong, separated	***P. solchi***
–	Trochanter apophysis long and rod-shaped, procursus with dorsal apophysis	**37**
37	Embolus straight, procursus with round dorsal apophysis and flat prolateral apophysis; epigynum elongate oval, anterior plate slightly curved posteriorly and bulged posterolaterally, anterior arch recurved, pore plates elongate oblong, separated	***P. seokmodoensis***
–	Embolus curved, procursus with serrated dorsal apophysis and hooked prolateral apophysis; epigynum elongate trapezoidal, anterior plate straight posteriorly, anterior arch strongly recurved, pore plates elongate ovoid, separated	***P. chilgapsanensis***
38	Trochanter apophysis long	**39**
–	Trochanter apophysis short	**47**
39	Trochanter apophysis finger-shaped	**40**
–	Trochanter apophysis rod-shaped	**41**
40	Embolus curved, procursus with curved and pointed prolateral apophysis; epigynum subcircular, anterior plate curved posteriorly, anterior arch sinuous, pore plates elongate oval, separated	***P. acutulus***
–	Embolus straight, procursus with membranous prolateral apophysis; epigynum elongate ovoid, anterior plate emarginate posteriorly, anterior arch sinuous, pore plates oblong, separated	***P. geogeum***
41	Trochanter apophysis with hair	**42**
–	Trochanter apophysis without hair	**44**
42	Embolus straight and much longer than uncus, uncus subquadrate; epigynum elongate octagonal, anterior plate emarginate posteriorly, anterior arch recurved, pore plates elongate obovate, separated	***P. yeongheung***
–	Embolus curved and slightly longer than uncus, uncus auriculate	**43**
43	Uncus proximally indented, procursus with slender dorsal apophysis and blunt-tipped ventral apophysis; epigynum urn-shaped, anterior plate slightly curved posteriorly, anterior arch short and recurved, pore plates elongate obovate, separated	***P. wonju***
–	Uncus proximally rounded, procursus with pointed prolatero-distal apophysis and claw-shaped retrolatero-distal apophysis; epigynum subcircular, anterior plate slightly curved posteriorly, anterior arch sinuous, pore plates oval, separated	***P. crassus***
44	Trochanter apophysis with upcurved tip, embolus slightly longer than uncus and curved	**45**
–	Trochanter apophysis with blunt or curled tip, embolus much longer than uncus and straight	**46**
45	Uncus subsquare, procursus with finger-shaped dorso-distal apophysis and claw-shaped ventro-distal apophysis; epigynum subcircular, anterior plate straight posteriorly, anterior arch sinuous, pore plates semicircular, separated	***P. yongin***
–	Uncus auriculate, procursus with finger-shaped dorso-distal apophysis and claw-shaped ventro-distal apophysis; epigynum subcircular, anterior plate straight posteriorly, anterior arch recurved, pore plates oval, separated	***P. suraksanensis***
46	Trochanter apophysis with blunt tip, procursus with pointed dorso-distal apophysis and pointed ventro-distal apophysis; epigynum ovoid, anterior plate slightly curved posteriorly, anterior arch straight, pore plates oval, separated	***P. worak***
–	Trochanter apophysis with curled tip, procursus with blunt dorso-distal apophysis and claw-shaped ventro-distal apophysis; epigynum elongate trapezoidal, anterior plate slightly curved posteriorly and bulged posterolaterally, anterior arch strongly recurved, pore plates oval, separated	***P. kwangkyosanensis***
47	Uncus rounded, procursus arcuate; epigynum subcircular, anterior plate curved posteriorly, anterior arch slightly emarginate, pore plates ovoid, separated	***P. pojeonensis***
–	Uncus trapezoid, quadrate or auriculate, procursus geniculate	**48**
48	Uncus trapezoid or quadrate	**49**
–	Uncus auriculate	**50**
49	Uncus trapezoid and triangular proximally, trochanter apophysis spatulate, embolus much longer than uncus and strongly curved; epigynum subcircular, anterior plate curved posteriorly, anterior arch recurved, pore plates oval, separated	***P. baegamsan* sp. nov**.
–	Uncus quadrate and blunt proximally, trochanter apophysis geniculate, embolus slightly longer than uncus and slightly curved; epigynum subcircular, anterior plate curved posteriorly, anterior arch sinuous, pore plates oval, separated	***P. pocheonensis***
50	Embolus spatulate; epigynum elongate oval, anterior plate curved posteriorly, anterior arch sinuous, pore plates reniform, separated	***P. gangneung***
–	Embolus virgate	**51**
51	Embolus straight	**52**
–	Embolus curved	**53**
52	Embolus slightly longer than uncus, uncus distally rounded-indented; epigynum subcircular, anterior plate slightly curved posteriorly, anterior arch recurved, pore plates oval, separated	***P. mohang***
–	Embolus much longer than uncus, uncus distally rounded-projecting; epigynum subcircular, anterior plate slightly curved posteriorly, anterior arch slightly recurved, pore plates ovoid, separated	***P. joreongensis***
53	Embolus strongly curved; epigynum subcircular, anterior plate strongly emarginate posteriorly, anterior arch recurved, pore plates obovate, separated	***P. namhae* sp. nov**.
–	Embolus slightly curved	**54**
54	Trochanter apophysis with upcurved tip; epigynum subrhomboidal, anterior plate curved posteriorly, anterior arch strongly recurved, pore plates rounded, separated	***P. unaksanensis***
–	Trochanter apophysis with blunt tip	**55**
55	Trochanter apophysis lobate; epigynum subcircular, anterior plate emarginate posteriorly, anterior arch slightly recurved, pore plates oval, separated	***P. nodong***
–	Trochanter apophysis finger-shaped or spatulate	**56**
56	Trochanter apophysis finger-shaped	**57**
–	Trochanter apophysis spatulate	**62**
57	Trochanter apophysis with hair	**58**
–	Trochanter apophysis without hair	**59**
58	Uncus distally indented, procursus with finger-shaped dorso-distal apophysis; epigynum subcircular, anterior plate sinuous posteriorly, anterior arch recurved, pore plates obovate, separated	***P. sokkrisanensis***
–	Uncus distally protuberant, procursus with claw-shaped dorso-distal apophysis; epigynum elongate ovoid, anterior plate curved posteriorly, anterior arch strongly recurved, pore plates ovoid, separated	***P. gochang***
59	Procursus with dorso-distal apophysis	**60**
–	Procursus without dorso-distal apophysis	**61**
60	Procursus with finger-shaped dorso-distal apophysis and blunt ventro-distal apophysis; epigynum subcircular, anterior plate straight posteriorly, anterior arch sinuous, pore plates elongate obovate, separated	***P. jeocheon***
–	Procursus with claw-shaped dorso-distal apophysis and lacking ventro-distal apophysis; epigynum subcircular, anterior plate curved posteriorly, anterior arch sinuous, pore plates triangular, separated	***P. piagolensis***
61	Procursus with angular ventro-distal apophysis, prolateral apophysis bifurcated and pointed; epigynum subcircular, anterior plate curved posteriorly, anterior arch sinuous, pore plates rounded, separated	***P. gosuensis***
–	Procursus without ventro-distal apophysis, prolateral apophysis membranous and pointed; epigynum subcircular, anterior plate slightly curved posteriorly, anterior arch sinuous, pore plates semicircular, separated	***P. juwangensis***
62	Uncus distally serrated	**63**
–	Uncus distally bifurcated or truncated	**64**
63	Procursus with blunt retrolatero-distal apophysis and branched prolateral apophysis; epigynum subcircular, anterior plate curved posteriorly, anterior arch slightly recurved, pore plates oval, separated	***P. muju***
–	Procursus with claw-shaped retrolatero-distal apophysis and curved prolateral apophysis; epigynum subcircular, anterior plate emarginate posteriorly, anterior arch sinuous, pore plates oval, separated	***P. extumidus***
64	Uncus distally bifurcated, procursus with claw-shaped dorso-distal apophysis and lacking dorsal apophysis; epigynum subcircular, anterior plate curved posteriorly, anterior arch sinuous, pore plates elongate ovoid, separated	***P. hongseong***
–	Uncus distally truncated, procursus without dorso-distal apophysis and with triangular dorsal apophysis; epigynum subcircular, anterior plate emarginate posteriorly, anterior arch straight, pore plates triangular, separated	***P. duryun***

### Species descriptions

#### Pholcus baegamsan sp. nov.

Taxon classificationAnimaliaAraneaePholcidae

92F87D7A-9400-5688-A607-B35E389369AE

https://zoobank.org/5570141C-73D9-4BA5-8BAB-D013AE3ADAC2

Figs 2, 6 A, 7A

##### Material examined.

***Holotype***. South Korea • ♂ (NIBR, #TTQXIV0000000271); Jeonbuk State; Sunchang-gun; Bokheung-myeon; Bongdeok-ri; Mt. Baegamsan; 35°27.898'N, 126°52.662'E; alt. 380 m; 25 September 2022; S.Y. Lee, C.M. Jang & S.T. Kim leg. ***Paratypes***. South Korea • ♀ (NIBR, #NIBRIV0000910317); • 2♂♂ (KKU, #KKU-ARA-Phol-20220925-01~02); • 2♀♀ (KKU, #KKU-ARA-Phol-20220925-03~04); same data as for holotype.

##### Diagnosis.

**Figure 2.**
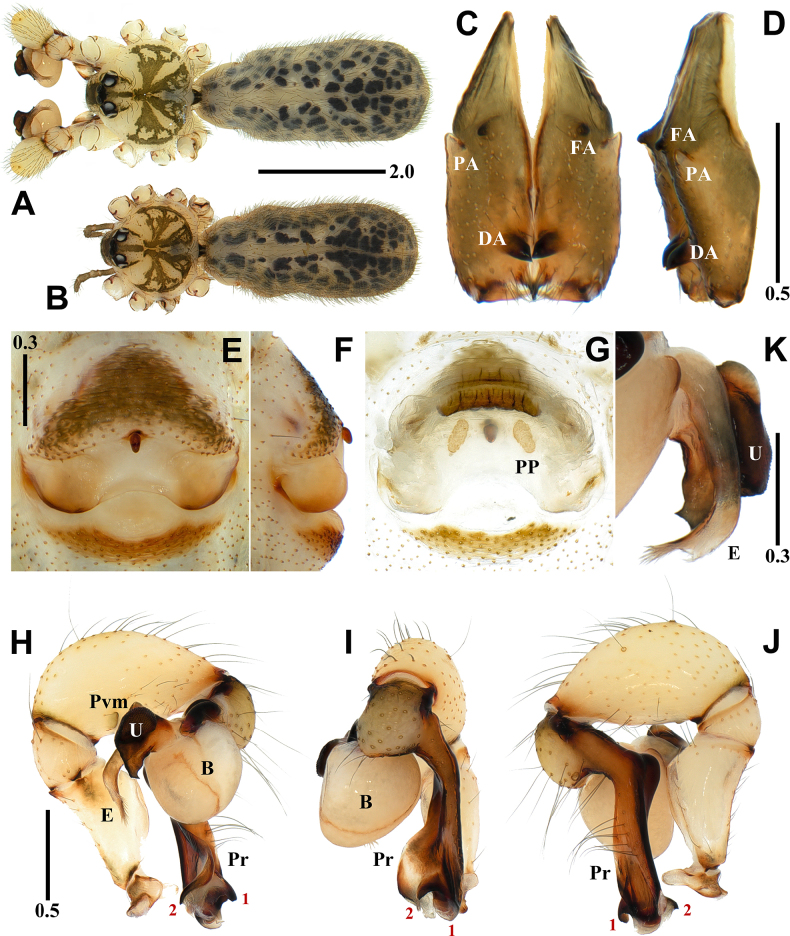
*Pholcus baegamsan* sp. nov. holotype male (**A, C, D, H–K**) and paratype female (**B, E–G**). **A, B**. Habitus (dorsal view); **C, D**. Chelicerae (**C**. Frontal view; **D**. Lateral view); **E, F**. Epigynum (**E**. Ventral view; **F**. Lateral view); **G**. Internal genitalia, dorsal view; **H–J**. Palp (1 = dorso-distal apophysis, 2 = prolateral apophysis); **K**. Bulbal processes. Abbreviations: B = bulb, DA = distal apophysis, E = embolus, FA = frontal apophysis, PA = proximo-lateral apophysis, PP = pore plate, Pr = procursus, Pvm = prolatero-ventral modification, U = uncus. Scale bars in mm.

*Pholcus baegamsan* sp. nov. is similar to *Pholcus duryun* Jang, Bae, Lee, Yoo & Kim, 2023 (Jang et al. 2023: fig. 2E–K), but can be distinguished from the latter by the combination of the following characteristics: Males – uncus with angular protrusions with a pointed tip distally and angular protrusion proximally (Fig. 2H); procursus with a dorso-distal apophysis blunt and bend prolaterally (numbered 1 in Fig. 2H–J) vs uncus with angular protrusions with a truncated tip distally and round protrusion proximally; procursus with a triangular dorso-distal apophysis. Females – anterior arch recurved and pore plates oval (Fig. 2G) vs anterior arch straight, pore plates triangular.

##### Description.

**Male** (holotype): Habitus as in Fig. 2A. Total length 5.43. Carapace: 1.72 long/1.79 wide. Eyes: ALE 0.18, AME 0.13, PLE 0.16, PME 0.16, ALE-AME 0.04, ALE-PLE contiguous, AME-AME 0.06, AME-PME 0.06, PLE-PME 0.04, PME-PME 0.26. Chelicera: 0.89 long/0.28 wide. Legs: I 46.87 (11.79, 0.75, 12.05, 19.86, 2.42), II 30.98 (8.38, 0.74, 7.89, 12.47, 1.50), III 21.04 (6.11, 0.66, 5.04, 8.14, 1.09), IV 28.22 (8.09, 0.61, 7.10, 11.18, 1.24), tibia I L/d 72. Abdomen: 3.57 long/1.62 wide.

Carapace pale yellowish brown, cephalic region with pale blackish brown median and marginal bands, thoracic region with pale blackish brown radial and marginal bands (Fig. 2A). Chelicera with three apophyses; blunt proximo-lateral apophysis diagonally upward and protruding out of chelicera, short and blunt frontal apophysis protruding forward, and thick and pointed distal apophysis diagonally downward (Fig. 2C, D). Legs yellowish brown, retrolateral trichobothrium on tibia I at 7% proximally, tarsus I with < 30 indistinct and irregular pseudosegments except 10 distally, leg formula I-II-IV-III. Abdomen cylindrical, pale and dull yellowish brown with an elongate, longitudinal cardiac pattern and numerous black irregular spots (Fig. 2A). Palp (Fig. 2H–K): trochanter apophysis ~0.24× as long as femur, spatulate, broad, distally rounded (Fig. 2J); palpal tibia with a rectangular prolatero-ventral modification (Fig. 2H); bulb pale yellowish brown, cordiform, appendix absent (Fig. 2H, K); uncus dark blackish brown with angular protrusions with a pointed tip distally and angular protrusion with a roundly granulated surface proximally, pseudo-appendix absent (Fig. 2H, K); embolus un-modified, weakly sclerotized with some semi-transparent distal fringed processes (Fig. 2H, K); procursus large and brown with dark blackish brown margin, distinct ventral knee present, strongly sclerotized, two apophyses present, dorso-distal apophysis blunt and bend prolaterally (numbered 1 in Fig. 2H–J), prolateral apophysis strongly curved ventrally (numbered 2 in Fig. 2H–J); a single long, translucent spine on the dorsal ridge (Fig. 6A).

**Female** (paratype): General appearance similar to male, habitus as in Fig. 2B. Total length 4.98. Carapace: 1.55 long/1.63 wide. Eyes: ALE 0.16, AME 0.10, PLE 0.14, PME 0.15, ALE-AME 0.04, ALE-PLE contiguous, AME-AME 0.06, AME-PME 0.06, PLE-PME 0.03, PME-PME 0.21. Legs: I 29.63 (7.46, 0.59, 7.67, 12.09, 1.82), II 20.92 (5.76, 0.62, 5.23, 8.05, 1.26), III 15.39 (4.33, 0.54, 3.72, 5.78, 1.02), IV 21.16 (6.28, 0.51, 5.22, 7.94, 1.21), tibia I L/d 42. Abdomen: 3.32 long/1.48 wide.

Epigynum (Fig. 2E, F): subcircular, length and width approximately subequal, anterior epigynal plate sclerotized and triangular, strongly recurved posteromedially with slightly sclerotized and bulged posterolateral margins, posterior epigynal plate narrow, knob short and thick with a blunt tip. Internal genitalia (Fig. 2G): anterior arch slightly recurved, pore plates oval, separated far from the anterior arch, separated far from each other, both margins membranous.

##### Variation.

Tibia I in three males (holotype and paratypes): 12.05, 10.03, 11.62. Tibia I in three females (paratypes): 7.67, 7.53, 8.20.

##### Habitat.

The species was hand-collected from rocky areas, such as rock walls and beneath rocks, in mountainous mixed forests (Fig. 1B).

##### Distribution.

Korea (Mt. Baegamsan, Sunchang-gun, Jeonbuk State) (Fig. 1A).

##### Etymology.

The specific name is a noun in apposition referring to the type locality, Mt. Baegamsan, Sunchang-gun, Jeonbuk State.

#### Pholcus hongcheon sp. nov.

Taxon classificationAnimaliaAraneaePholcidae

269B0F1D-427A-5EDC-ADF3-E8CAC1B81D7B

https://zoobank.org/086FAC2E-173D-4E5D-A654-052F672432BC

Figs 3, 6 B, 7B

##### Material examined.

***Holotype***. South Korea • ♂ (NIBR, #TTQXIV0000000272); Gangwon-do; Hongcheon-gun; Hwachon-myeon; Yasidae-ri; Mt. Garisan; 37°49.484'N, 127°57.305'E; alt. 280 m; 29 July 2023, C.M. Jang & S.T. Kim leg. ***Paratypes***. South Korea • ♀ (NIBR, #NIBRIV0000910315); same data as for holotype • 2♂♂ (KKU, #KKU-ARA-Phol-20230908-01~02); • 2♀♀ (KKU, #KKU-ARA-Phol-20230908-03~04); same locality as for holotype; 8 September 2023; C.M. Jang & S.T. Kim leg.

##### Diagnosis.

**Figure 3.**
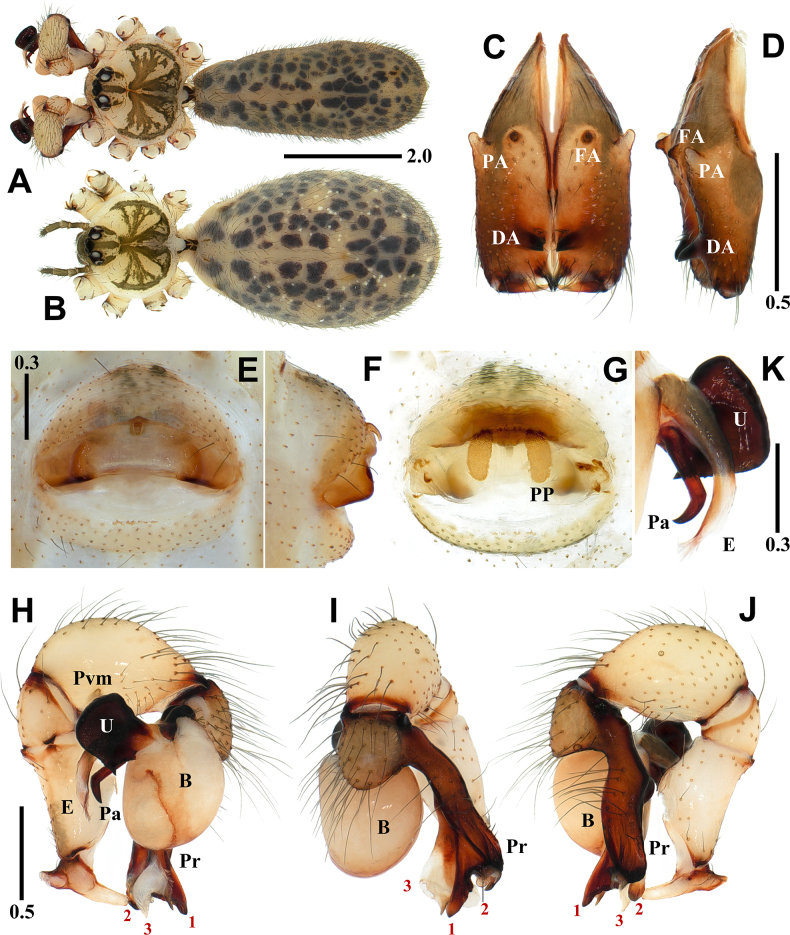
*Pholcus hongcheon* sp. nov. holotype male (**A, C, D, H–K**) and paratype female (**B, E–G**). **A, B**. Habitus (dorsal view); **C, D**. Chelicerae (**C**. Frontal view; **D**. Lateral view); **E, F**. Epigynum (**E**. Ventral view; **F**. Lateral view); **G**. Internal genitalia, dorsal view; **H–J**. Palp (1 = dorso-distal apophysis, 2 = ventro-distal apophysis, 3 = prolateral apophysis); **K**. Bulbal processes. Abbreviations: B = bulb, DA = distal apophysis, E = embolus, FA = frontal apophysis, PA = proximo-lateral apophysis, Pa = pseudo-appendix, PP = pore plate, Pr = procursus, Pvm = prolatero-ventral modification, U = uncus. Scale bars in mm.

*Pholcus hongcheon* sp. nov. is similar to *Pholcus deokjeok* Jang, Yoo, Kim & Bae, 2025 (Jang et al. 2025a: fig. 2e–k), but can be distinguished from the latter by the combination of the following characteristics: Males – uncus square with a pointed tip distally, pseudo-appendix thick and sickle-shaped (Fig. 3H, K); procursus with three apophyses, dorso-distal apophysis large and thick (numbered 1 in Fig. 3H–J), ventro-distal apophysis round (numbered 2 in Fig. 3H–J), retrolatero-distal apophysis absent, vs uncus rounded square without a pointed tip distally, two pseudo-appendices thick and slightly curved; procursus with four apophyses, dorso-distal apophysis large and slender, ventro-distal apophysis large and claw-shaped, retrolatero-distal apophysis present.

Females – anterior epigynal plate semi-circular and slightly recurved posteriorly, with posterolateral margins slightly sclerotized and protruding, posterior epigynal plate narrow, knob short, unsclerotized (Fig. 3E, F) vs anterior epigynal plate triangular and sinuous posteriorly, posterolateral margins slightly sclerotized and not protruding, posterior epigynal plate broad, knob very short, sclerotized; anterior arch slightly recurved, pore plates large and elongate oval (Fig. 3G) vs anterior arch straight, pore plates semicircular.

##### Description.

**Male** (holotype): Habitus as in Fig. 3A. Total length 5.51. Carapace: 1.65 long/1.83 wide. Eyes: ALE 0.18, AME 0.12, PLE 0.17, PME 0.16, ALE-AME 0.06, ALE-PLE contiguous, AME-AME 0.05, AME-PME 0.06, PLE-PME 0.07, PME-PME 0.25. Legs: I 47.60 (11.85, 0.71, 12.18, 20.30, 2.56), II 31.45 (8.53, 0.72, 7.87, 12.67, 1.66), III 21.74 (6.30, 0.62, 5.37, 8.27, 1.18), IV 28.60 (8.35, 0.60, 7.09, 11.12, 1.44), tibia I L/d 66. Abdomen: 3.72 long/1.59 wide.

Carapace pale yellowish brown, cephalic region with pale blackish brown median and marginal bands, thoracic region with pale blackish brown radial and marginal bands (Fig. 3A). Chelicera with three apophyses; blunt proximo-lateral apophysis diagonally upward and protruding out of chelicera, short and blunt frontal apophysis protruding forward, and thick and pointed distal apophysis diagonally downward (Fig. 3C, D). Legs yellowish brown, retrolateral trichobothrium on tibia I at 5% proximally, tarsus I with < 40 indistinct and irregular pseudosegments except 12 distally, leg formula I-II-IV-III. Abdomen cylindrical, pale and dull yellowish brown with an elongate, longitudinal cardiac pattern and numerous black irregular spots (Fig. 3A). Palp (Fig. 3H–K): trochanter apophysis ~0.42× as long as femur, straight, slender, distally blunt (Fig. 3H, J); palpal tibia with a rectangular prolatero-ventral modification (Fig. 3H); bulb pale yellowish brown, cordiform, appendix absent (Fig. 3H, K); uncus dark blackish brown and square with a pointed tip distally, pseudo-appendix thick and sickle-shaped (Fig. 3H, K); embolus unmodified, weakly sclerotized with some semi-transparent distal fringed processes (Fig. 3H, K); procursus large and brown with dark blackish brown margin, distinct ventral knee present, strongly sclerotized, three apophyses present, dorso-distal apophysis large and triangular (numbered 1 in Fig. 3H–J), ventro-distal apophysis round (numbered 2 in Fig. 3H–J), prolatero-distal apophysis membranous with a pointed tip (numbered 3 in Fig. 3H–J); a single stout, black spine on the dorsal ridge (Fig. 6B).

**Female** (paratype): General appearance similar to male, habitus as in Fig. 3B. Total length 5.83. Carapace: 1.64 long/1.75 wide. Eyes: ALE 0.16, AME 0.11, PLE 0.16, PME 0.14, ALE-AME 0.06, ALE-PLE contiguous, AME-AME 0.05, AME-PME 0.05, PLE-PME 0.04, PME-PME 0.21. Legs: I 33.98 (8.42, 0.66, 8.64, 14.04, 2.22), II 23.50 (6.43, 0.68, 5.92, 9.21, 1.26), III 17.05 (4.91, 0.61, 4.20, 6.34, 0.99), IV 22.86 (6.72, 0.59, 5.84, 8.58, 1.13), tibia I L/d 49. Abdomen: 3.89 long/2.54 wide.

Epigynum (Fig. 3E, F): subcircular, length and width approximately subequal, anterior epigynal plate semi-circular and unsclerotized and slightly recurved posteriorly, posterolateral margins slightly sclerotized and protruding, posterior epigynal plate narrow, knob short, small, unsclerotized with a blunt tip. Internal genitalia (Fig. 3G): anterior arch slightly recurved, pore plates large and elongate oval, contiguous with the anterior arch, separated far from each other, both margins membranous.

##### Variation.

Tibia I in three males (holotype and paratypes): 12.18, 14.37, 13.49. Tibia I in three females (paratypes): 8.64, 8.90, 8.93.

##### Habitat.

The species was hand-collected from rocky areas, such as rock walls and beneath rocks, in mountainous mixed forests (Fig. 1C).

##### Distribution.

Korea (Mt. Garisan, Hongcheon-gun, Gangwon-do) (Fig. 1A).

##### Etymology.

The specific name is a noun in apposition referring to the type locality, Hongcheon-gun, Gangwon-do.

#### Pholcus namhae sp. nov.

Taxon classificationAnimaliaAraneaePholcidae

36177567-EC35-57DD-8990-9CA9C78221E1

https://zoobank.org/20222F64-8632-4D87-852B-C50E04064D62

Figs 4, 6 C, 7C

##### Material examined.

***Holotype***. South Korea • ♂ (NIBR, #TTQXIV0000000273); Gyeongsangnam-do; Namhae-gun; Sangju-myeon; Sangju-ri; Boriam Temple; 34°45.138'N, 127°58.941'E; alt. 650 m; 7 September 2023; C.M. Jang & S.T. Kim leg. ***Paratypes***. South Korea • ♀ (NIBR, #NIBRIV0000910312); same data as for holotype • 2♂♂ (KKU, #KKU-ARA-Phol-20190820-01), • 2♀♀ (KKU, #KKU-ARA-Phol-20190820-02); same locality as for holotype; 20 August 2019; S.T. Kim & S.Y. Lee leg.

##### Diagnosis.

**Figure 4.**
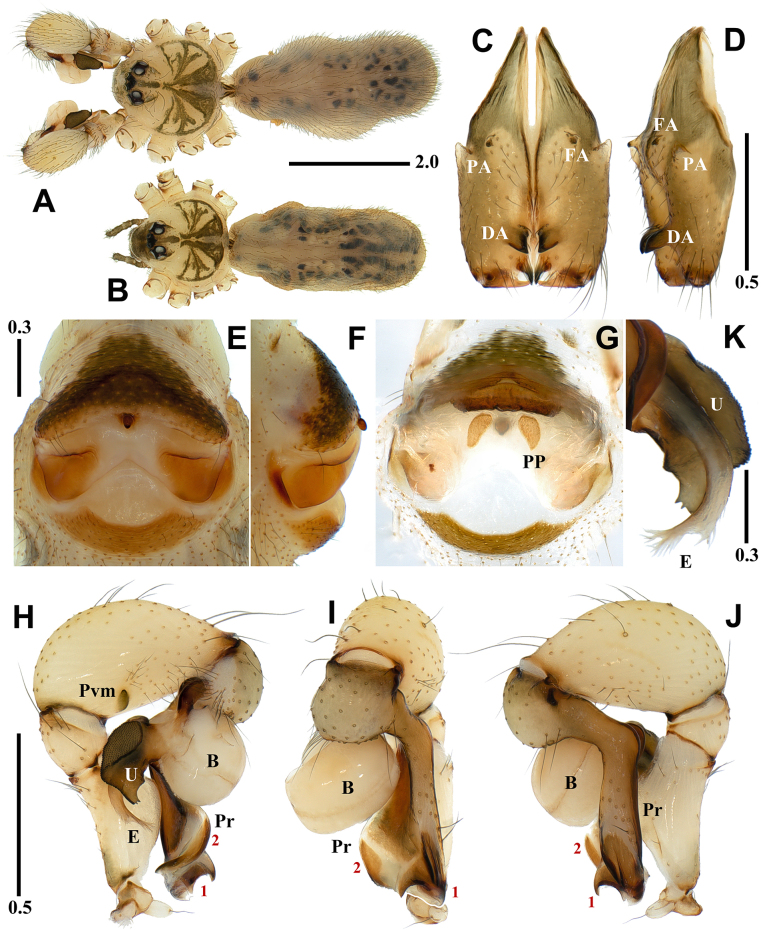
*Pholcus namhae* sp. nov. holotype male (**A, C, D, H–K**) and paratype female (**B, E–G**). **A, B**. Habitus (dorsal view); **C, D**. Chelicerae (**C**. Frontal view; **D**. Lateral view); **E, F**. Epigynum (**E**. Ventral view; **F**. Lateral view); **G**. Internal genitalia, dorsal view; **H–J**. Palp (1 = dorso-distal apophysis, 2 = prolateral apophysis); **K**. Bulbal processes. Abbreviations: B = bulb, DA = distal apophysis, E = embolus, FA = frontal apophysis, PA = proximo-lateral apophysis, PP = pore plate, Pr = procursus, Pvm = prolatero-ventral modification, U = uncus. Scale bars in mm.

*Pholcus namhae* sp. nov. is similar to *Pholcus palgongensis* Seo, 2014 (Seo 2014: figs 1R, S, 5A–D, G, H), but can be distinguished from the latter by the combination of the following characteristics: Males – uncus with a trifurcated distal tip and a round proximal tip (Fig. 4H, K); procursus with a large prolateral apophysis protruding markedly outward from the procursus (numbered 2 in Fig. 4H–J) vs uncus with a blunt non-trifurcated distal tip and lacking a proximal tip; procursus with a prolateral apophysis not protruding outward from the procursus. Females – anterior epigynal plate strongly emarginate posteromedially, knob short (Fig. 4E, F) vs anterior epigynal plate recurved posteromedially, knob moderately long; pore plates obovate, longitudinal (Fig. 4G) vs pore plates oval, horizontal.

##### Description.

**Male** (holotype): Habitus as in Fig. 4A. Total length 5.16. Carapace: 1.76 long/1.69 wide. Eyes: ALE 0.19, AME 0.11, PLE 0.17, PME 0.14, ALE-AME 0.04, ALE-PLE contiguous, AME-AME 0.08, AME-PME 0.11, PLE-PME 0.05, PME-PME 0.27. Legs: I 40.67 (10.14, 0.75, 10.17, 17.22, 2.39), II 27.41 (7.47, 0.74, 6.76, 10.90, 1.54), III 19.40 (5.60, 0.61, 4.70, 7.28, 1.21), IV 25.65 (7.33, 0.62, 6.42, 9.85, 1.43), tibia I L/d 56. Abdomen: 3.20 long/1.61 wide.

Carapace pale yellowish brown, cephalic region with pale blackish brown median and marginal bands, thoracic region with pale blackish brown radial and marginal bands (Fig. 4A). Chelicera with three apophyses; blunt proximo-lateral apophysis diagonally upward and protruding out of chelicera, short and blunt frontal apophysis protruding forward, and thick and pointed distal apophysis diagonally downward (Fig. 4C, D). Legs yellowish brown, retrolateral trichobothrium on tibia I at 5% proximally, tarsus I with < 35 indistinct and irregular pseudosegments except 10 distally, leg formula I-II-IV-III. Abdomen cylindrical, pale and dull reddish brown with an elongate, longitudinal cardiac pattern and numerous black irregular spots (Fig. 4A). Palp (Fig. 4H–K): trochanter apophysis ~1/4 as long as femur, stubby, broad, distally rounded with a hair (Fig. 4H, J); palpal tibia with an oval prolatero-ventral modification (Fig. 4H); bulb pale yellowish brown, cordiform, appendix absent (Fig. 4H, K); uncus dull blackish brown with a trifurcated tip distally and a round tip proximally, medial surface granulated, pseudo-appendix absent (Fig. 4H, K); embolus unmodified, weakly sclerotized with some semi-transparent distal fringed processes (Fig. 4H, K); procursus large and pale brown with dark blackish brown margin, distinct ventral knee present, moderately sclerotized, two apophyses present, dorso-distal apophysis large and claw-shaped (numbered 1 in Fig. 4H–J), prolateral apophysis large with a pointed tip and protruding markedly outward from the procursus (numbered 2 in Fig. 4H–J); a single stout, black spine on the dorsal ridge (Fig. 6C).

**Female** (paratype): General appearance similar to male, habitus as in Fig. 4B. Total length 4.96. Carapace: 1.64 long/1.72 wide. Eyes: ALE 0.18, AME 0.10, PLE 0.17, PME 0.15, ALE-AME 0.04, ALE-PLE contiguous, AME-AME 0.06, AME-PME 0.08, PLE-PME 0.04, PME-PME 0.21. Legs: I 29.08 (7.30, 0.63, 7.32, 11.67, 2.16), II 19.89 (5.54, 0.57, 4.98, 7.45, 1.35), III 14.83 (4.35, 0.54, 3.53, 5.39, 1.02), IV 20.17 (5.82, 0.54, 5.09, 7.51, 1.21), tibia I L/d 46. Palp: Abdomen: 3.30 long/1.41 wide.

Epigynum (Fig. 4E, F): subcircular, longer than wide, anterior epigynal plate strongly emarginate posteromedially with sclerotized posterolateral margins, posterior epigynal plate narrow, knob very short and thick with a blunt tip. Internal genitalia (Fig. 4G): anterior arch triangularly recurved, pore plates obovate, pointed oval, vertical, separated far from the anterior arch, separated far from each other, both margins membranous.

##### Variation.

Tibia I in three males (holotype and paratypes): 10.17, 11.37, 10.49. Tibia I in three females (paratypes): 7.32, 7.70, 7.64.

##### Habitat.

The species was hand-collected from rocky areas, such as rock walls and beneath rocks, in mountainous mixed forests (Fig. 1D).

##### Distribution.

Korea (Boriam Temple, Namhae-gun, Gyeongsangnam-do) (Fig. 1A).

##### Etymology.

The specific name is a noun in apposition referring to the type locality, Namhae-gun, Gyeongsangnam-do.

#### Pholcus yeongcheon sp. nov.

Taxon classificationAnimaliaAraneaePholcidae

1645359D-CE73-5D89-B62E-ACA8F7F876C6

https://zoobank.org/6CA623FE-9BF6-44F9-855D-EA50D51E4ACE

Figs 5, 6 D, 7D

##### Material examined.

***Holotype***. South Korea • ♂ (NIBR, #TTQXIV0000000274); Gyeongsangbuk-do; Yeongcheon-si; Sinnyeong-myeon; Hwanam-ri; Mt. Hwasan; 36°05.460'N, 128°46.867'E; alt. 740 m; 8 September 2023; C.M. Jang & S.T. Kim leg. ***Paratypes***. South Korea • ♀ (NIBR, #NIBRIV0000910314); • 2♂♂ (KKU, #KKU-ARA-Phol-20230908-05~06); • 2♀♀ (KKU, #KKU-ARA-Phol-20230908-07~08); same data as for holotype.

##### Diagnosis.

**Figure 5.**
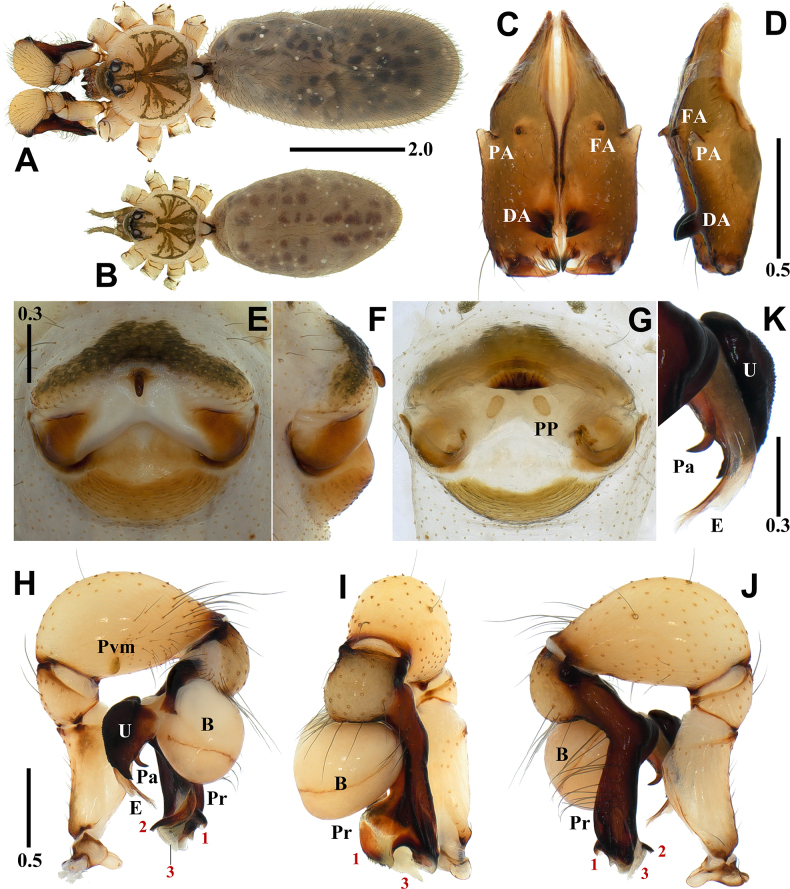
*Pholcus yeongcheon* sp. nov. holotype male (**A, C, D, H–K**) and paratype female (**B, E–G**). **A, B**. Habitus (dorsal view); **C, D**. Chelicerae (**C**. Frontal view; **D**. Lateral view); **E, F**. Epigynum (**E**. Ventral view; **F**. Lateral view); **G**. Internal genitalia, dorsal view; **H–J**. Palp (1 = dorso-distal apophysis, 2 = prolateral apophysis, 3 = ventral membranous process); **K**. Bulbal processes. Abbreviations: B = bulb, DA = distal apophysis, E = embolus, FA = frontal apophysis, PA = proximo-lateral apophysis, Pa = pseudo-appendix, PP = pore plate, Pr = procursus, Pvm = prolatero-ventral modification, U = uncus. Scale bars in mm.

**Figure 6.**
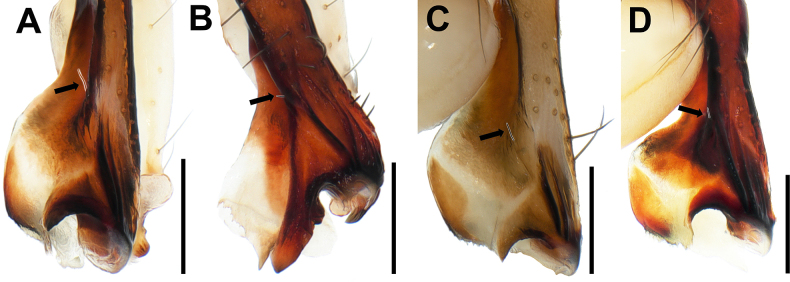
Dorsal spines. **A**. *Pholcus baegamsan* sp. nov.; **B**. *Pholcus hongcheon* sp. nov.; **C**. *Pholcus namhae* sp. nov.; **D**. *Pholcus yeongcheon* sp. nov. (arrow points to a dorsal spine). Scale bars: 0.3 mm.

*Pholcus yeongcheon* sp. nov. is similar to *Pholcus gajiensis* Seo, 2014 (Seo 2014: figs 1H, I, 3A–D, G, H), but can be distinguished from the latter by the combination of the following characteristics: Males – uncus with a finely serrated edge, pseudo-appendix slender and hook-shaped (Fig. 5H, K); procursus with a large claw-shaped dorso-distal apophysis (numbered 1 in Fig. 5H–J), a single translucent dorsal spine present (Fig. 6D) vs uncus with a coarsely serrated edge, pseudo-appendix thick and hook-shaped; procursus with a small claw-shaped dorso-distal apophysis, a single black and rigid dorsal spine present. Females – posterior epigynal plate broad; pore plates oval (Fig. 5G) vs posterior epigynal plate narrow; pore plates elongate oval.

**Figure 7.**
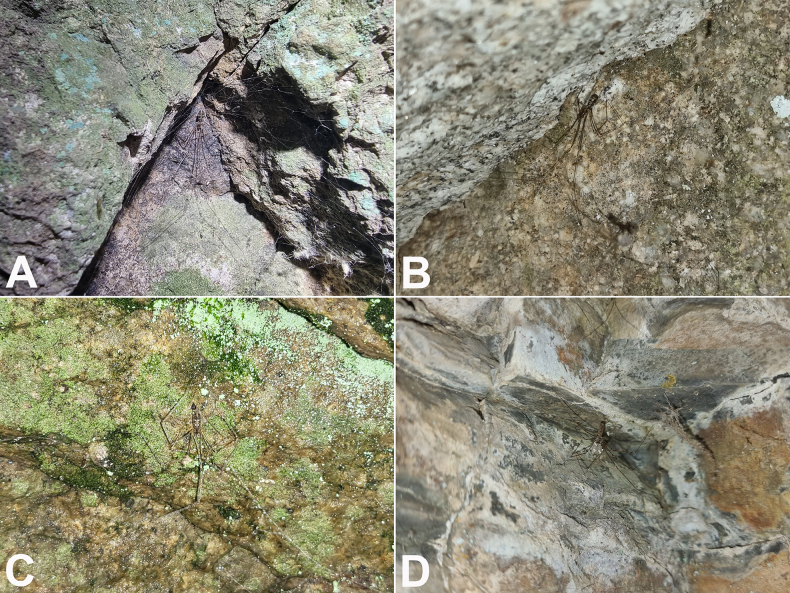
Habitus of the new species (female). **A**. *Pholcus baegamsan* sp. nov.; **B**. *Pholcus hongcheon* sp. nov.; **C**. *Pholcus namhae* sp. nov.; **D**. *Pholcus yeongcheon* sp. nov.

##### Description.

**Male** (holotype): Habitus as in Fig. 5A. Total length 6.63. Carapace: 1.92 long/1.91 wide. Eyes: ALE 0.17, AME 0.15, PLE 0.17, PME 0.12, ALE-AME 0.04, ALE-PLE contiguous, AME-AME 0.06, AME-PME 0.07, PLE-PME 0.04, PME-PME 0.26. Legs: I 47.58 (12.17, 0.77, 12.32, 20.00, 2.32), II 32.62 (8.98, 0.74, 8.29, 13.04, 1.57), III 22.13 (6.44, 0.68, 5.40, 8.42, 1.19), IV 29.25 (8.33, 0.69, 7.47, 11.32, 1.44), tibia I L/d 62. Abdomen: 4.45 long/2.25 wide.

Carapace pale reddish brown, cephalic region with pale blackish brown median and marginal bands, thoracic region with pale blackish brown radial and marginal bands (Fig. 5A). Chelicera with three apophyses; blunt proximo-lateral apophysis diagonally upward and protruding out of chelicera, short and pointed frontal apophysis protruding forward, and thick and pointed distal apophysis diagonally downward (Fig. 5C, D). Legs yellowish brown, retrolateral trichobothrium on tibia I at 6% proximally, tarsus I with < 30 indistinct and irregular pseudosegments except 15 distally, leg formula I-II-IV-III. Abdomen cylindrical, pale and dull grayish brown with an elongate, longitudinal cardiac pattern and numerous black irregular spots (Fig. 5A). Palp (Fig. 5H–K): trochanter apophysis ~0.14× as long as femur, stubby, broad, distally rounded (Fig. 5H, J); bulb pale yellowish brown, cordiform, appendix absent (Fig. 5H, K); uncus dull blackish brown with a pointed tip distally and a round tip proximally, edge finely serrated, pseudo-appendix slender and hook-shaped (Fig. 5H, K); embolus unmodified, weakly sclerotized with some semi-transparent distal fringed processes (Fig. 5H, K); procursus large and dark brown with dark blackish brown margin, distinct ventral knee present, strongly sclerotized, two apophyses and present, dorso-distal apophysis small and claw-shaped (numbered 1 in Fig. 5H–J), prolateral apophysis large and curved with a truncated tip (numbered 2 in Fig. 5H–J) and a ventral membranous process; a single short, translucent spine on the dorsal ridge (Fig. 6D).

**Female** (paratype): General appearance similar to male, habitus as in Fig. 5B. Total length 5.13. Carapace: 1.42 long/1.36 wide. Eyes: ALE 0.15, AME 0.08, PLE 0.14, PME 0.14, ALE-AME 0.05, ALE-PLE contiguous, AME-AME 0.06, AME-PME 0.07, PLE-PME 0.05, PME-PME 0.20. Legs: I 25.94 (6.60, 0.57, 6.52, 10.40, 1.85), II 18.11 (4.97, 0.57, 4.47, 6.84, 1.26), III 13.41 (3.88, 0.47, 3.28, 4.85, 0.93), IV 17.79 (5.23, 0.51, 4.44, 6.62, 0.99), tibia I L/d 40. Abdomen: 3.43 long/2.00 wide.

Epigynum (Fig. 5E, F): subcircular, length and width approximately subequal, anterior epigynal plate strongly emarginate posteromedially with sclerotized posterolateral margins, posterior epigynal plate broad, knob moderately long and thick with a blunt tip. Internal genitalia (Fig. 5G): anterior arch recurved, pore plates oval, separated far from the anterior arch, separated far from each other, both margins sclerotized.

##### Variation.

Tibia I in three males (holotype and paratypes): 12.32, 10.37, 11.34. Tibia I in three females (paratypes): 6.52, 7.60, 7.93.

##### Habitat.

The species was hand-collected from rocky areas, such as rock walls and beneath rocks, in mountainous mixed forests (Fig. 1E).

##### Distribution.

Korea (Mt. Hwasan, Yeongcheon-si, Gyeongsangbuk-do) (Fig. 1A).

##### Etymology.

The specific name is a noun in apposition referring to the type locality, Yeongcheon-si, Gyeongsangbuk-do.

## Discussion

The genus *Pholcus* Walckenaer, 1805 is one of the most species-rich spider groups in South Korea, currently represented by three species groups: the *crypticolens* species group (4 species) (Paik 1978a; Kim et al. 2015), the *phalangioides* species group (1 species) (Paik 1978b), and the *phungiformes* species group (65 species, including the new species). However, several species belonging to the *phungiformes* species group that were previously poorly described, such as *P. kwangkyosanensis* Kim & Park, 2009 and *P. uksuensis* Kim & Ye, 2014, which have recently been redescribed or revalidated (Jang and Kim 2024; Jang et al. 2025c). Nevertheless, several species within this group still appear to require redescription. Paik (1978b) described six new species in this group: *P. acutulus* Paik, 1978; *P. crassus* Paik, 1978; *P. extumidus* Paik, 1978; *P. montanus* Paik, 1978; *P. socheunensis* Paik, 1978; and *P. sokkrisanensis* Paik, 1978. In his original descriptions, though, prolateral views were not provided in the illustrations, making it difficult to examine the uncus, one of the key bulbal processes. The shape of the uncus is a crucial diagnostic character of the male palp, and according to Lee et al. (2024), the structure of the male palpal uncus was relatively consistent among conspecific individuals. In particular, Huber (2011) noted that the specimens used in the description of *P. montanus* are extremely similar to the drawings in the original description by Paik (1978b). Even so, he emphasized the need for direct comparison of specimens and stated that the assignment of the specimens listed below to *P. montanus* is tentative. A comparison of the drawings presented in the original descriptions by Huber and Paik reveals that, while the retrolateral view of the male palp appears similar, the female epigynum looks entirely different, and the internal genitalia also show some differences. The authors collected numerous male and female specimens from several localities, including Mt. Odaesan, Pyeongchang-gun, Gangwon-do, which correspond well with Huber’s description. However, no specimens matching Paik’s description have been found to date. Nevertheless, based on the currently available evidence, the male and female specimens tentatively assigned to *P. montanus* by Huber appear to be conspecific, and it is possible that this species represents a new species distinct from *P. montanus*. Accordingly, to confirm this with certainty, a comparative morphological study with the holotype is required.

In addition, *P. pojeonensis* Kim & Yoo, 2008 and *P. deunggolensis* Kim & Kim, 2016 were poorly described and accompanied by low-resolution morphological photographs and illustrations (Kim and Yoo 2008; Kim and Kim 2016). Therefore, a detailed redescription of these species is required to clarify their taxonomic status. Of the 65 species (including the new species herein) belonging to the *Pholcus phungiformes* species group described from South Korea thus far, 64 are considered endemic, while *P. extumidus* Paik, 1978 has additionally been recorded from Japan. According to Irie (2009), *P. extumidus* was reported from Tsushima Island, Japan. However, this species has not yet been found on any South Korean islands, and given the brevity of his description and the limited information on the morphology of the genital organs, the identity of the specimens remains uncertain.

The *Pholcus phungiformes* species group exhibits an exceptionally high degree of regional endemism in the South Korea, with 64 of the 65 species being endemic to the region. While previous studies on pholcid spiders (Eberle et al. 2018; Meng et al. 2026) emphasized the pivotal role of microhabitat shifts, such as transitions between ground, space, and leaf-dwelling lifestyles, in driving diversification, the habitat data compiled from South Korea suggest that diversification in this group appears to be driven mainly by geographic isolation rather than microhabitat differentiation. Most species occupy broadly similar habitats, such as rock walls, road drains, and cave entrances, indicating a high degree of habitat similarity among species (Lee et al. 2021a; Yao et al. 2021). In addition, many species appear to exhibit strong site fidelity, remaining associated with specific micro-sites over time. As low dispersers, they are likely to experience long-term isolation among populations in different mountain regions. Such ecological similarity, combined with strong site fidelity, may contribute to morphological conservatism, with divergence largely restricted to the genitalia.

The rugged topography of South Korea is characterized by complex mountainous terrain, with approximately 70% of the land consisting of mountains and numerous valleys separating adjacent mountain systems, including major mountain ranges such as the Taebaek and Sobaek mountains (National Geographic Information Institute 2016; Korea Forest Service 2021). In such a landscape, even relatively small geographic barriers may be sufficient to restrict gene flow between populations. This pattern is consistent with small-scale endemism observed in other *Pholcus* radiations, such as those in the Canary Islands (Dimitrov and Ribera 2007; Dimitrov et al. 2008; Huber 2011).

Forests generally harbor a remarkably diverse array of wildlife species, including mammals, birds, and arthropods, and represent major terrestrial reservoirs of global biodiversity (Xofis et al. 2023). In South Korea, mountainous temperate mixed forests composed of both deciduous broadleaf and coniferous trees provide abundant rocky microhabitats suitable for spiders of the *Pholcus phungiformes* species group. These conditions may further promote the persistence of locally isolated populations across mountainous regions.

The discovery of four new species in this study indicates that the currently recognized diversity of Korean *Pholcus* does not fully reflect the actual species richness. Given that new species continue to be identified within a species-rich group, additional, as yet undocumented species are likely to remain undiscovered. Future studies integrating morphological and molecular data will be essential to better understand species boundaries and evolutionary relationships within this complex and species-rich group.

## Supplementary Material

XML Treatment for Pholcus

XML Treatment for Pholcus baegamsan

XML Treatment for Pholcus hongcheon

XML Treatment for Pholcus namhae

XML Treatment for Pholcus yeongcheon

## Additional information

### Artificial Intelligence (AI) use

The authors accept full responsibility for the content of the manuscript, including the disclosure of any use of AI.

No AI tools were used in the preparation of this manuscript.

### Author ORCIDs

C.M. Jang https://orcid.org/0000-0003-4343-2279

J.S. Yoo https://orcid.org/0000-0002-3243-2006

S.Y. Lee https://orcid.org/0000-0002-2066-3747

S.T. Kim https://orcid.org/0000-0001-5820-9294

### Data availability

All of the data that support the findings of this study are available in the main text.

## References

[B1] Dimitrov D, Ribera C (2007) The genus *Pholcus* (Araneae, Pholcidae) in the Canary Islands. Zoological Journal of the Linnean Society 151(1): 59–114. 10.1111/j.1096-3642.2007.00316.x

[B2] Dimitrov D, Arnedo M, Ribera C (2008) Colonization and diversification of the spider genus *Pholcus* Walckenaer, 1805 (Araneae, Pholcidae) in the Macaronesian archipelagos: Evidence for long-term occupancy yet rapid recent speciation. Molecular Phylogenetics and Evolution 48(2): 596–614. 10.1016/j.ympev.2008.04.027 18524631

[B3] Eberle J, Dimitrov D, Valdez-Mondragón A, Huber BA (2018) Microhabitat change drives diversification in pholcid spiders. BMC Evolutionary Biology 18: 141. 10.1186/s12862-018-1244-8 PMC6145181 30231864

[B4] Huber BA (2011) Revision and cladistic analysis of *Pholcus* and closely related taxa (Araneae, Pholcidae). Bonner Zoologische Monographien 58: 1–509.

[B5] Huber BA, Eberle J, Dimitrov D (2018) The phylogeny of pholcid spiders: a critical evaluation of relationships suggested by molecular data (Araneae, Pholcidae). ZooKeys 789: 51–101. 10.3897/zookeys.789.22781 PMC6193417 30344435

[B6] Irie T (2009) Pholcidae. In: Ono H (Ed.) The Spiders of Japan with Keys to the Families and Genera and Illustrations of the Species. Tokai University Press, Kanagawa, 106–111.

[B7] Jang CM, Kim ST (2024) Redescription of a poorly known spider, *Pholcus kwangkyosanensis* Kim & Park, 2009 (Araneae: Pholcidae) from Korea. Korean Journal of Environmental Biology 42(2): 172–175. 10.11626/KJEB.2024.42.2.172

[B8] Jang CM, Bae YS, Lee SY, Yoo JS, Kim ST (2023) Five new species of the *Pholcus phungiformes* species group (Araneae, Pholcidae) from South Korea. ZooKeys 1178: 97–114. 10.3897/zookeys.1178.104780 PMC10843697 38318118

[B9] Jang CM, Yoo JS, Kim ST, Bae YS (2025a) Rocky area inhabiting daddy long-legs spiders, *Pholcus* Walckenaer, 1805 (Araneae: Pholcidae) in mountainous mixed forests from South Korea. MDPI, Basel, Beijing, Wuhan, Barcelona, Belgrade, Novi Sad, Cluj, Manchester, 346–354. [printed version of: Forests 14(538): 1–8, 2023] 10.3390/books978-3-7258-3296-5

[B10] Jang CM, Bae YS, Kim ST (2025b) Two new spider species of the genus *Pholcus* Walckenaer, 1805 (Araneae, Pholcidae) from Korea. Korean Journal of Environmental Biology 42(4) [2024]: 541–547. [publ. in January 2025] 10.11626/KJEB.2024.42.4.541

[B11] Jang CM, Yoo JS, Kim ST (2025c) Description of one new *Pholcus* spider species and revalidation of *Pholcus uksuensis* Kim & Ye, 2014 (Araneae: Pholcidae) from Korea. Korean Journal of Environmental Biology 43(1): 77–83. 10.11626/KJEB.2025.43.1.077

[B12] Khmelik VV, Kozub D, Glazunov A (2006) Helicon Focus. Version 8.3.7. http://www.heliconsoft.com/heliconfocus.html [accessed 10 November 2025]

[B13] Kim BW, Lee W (2004) A new species of *Pholcus* (Araneae: Pholcidae) from Gosu Cave, Korea. Korean Journal of Systematic Zoology 20: 79–85.

[B14] Kim JP, Kim TW (2016) One new species of the genus *Pholcus* Walckenaer, 1805 (Araneae: Pholcidae) from Korea. Korean Arachnology 32(2): 13–20.

[B15] Kim JP, Park YC (2009) One new species of genus *Pholcus* (Araneae: Pholcidae) from Korea. Korean Arachnology 25: 99–103.

[B16] Kim JP, Ye SH (2014) A new species of the genus *Pholcus* Walckenaer, 1805 (Araneae: Pholcidae) from Korea. Korean Arachnology 30(1): 51–59.

[B17] Kim JP, Ye SH (2015) A new species of the genus *Pholcus* Walckenaer, 1805 (Araneae: Pholcidae) from Korea. Korean Arachnology 31(2): 73–80.

[B18] Kim JP, Yoo SH (2008) One new species of genus *Pholcus* (Araneae: Pholcidae) from Korea. Korean Arachnology 24: 1–6.

[B19] Kim JP, Lee JH, Lee JG (2015) Two new species of the genus *Pholcus* Walckenaer, 1805 (Araneae: Pholcidae), with redescription of *P. zichyi* Kulczyński, 1901 from Korea. Korean Arachnology 31(2): 53–71.

[B20] Korea Forest Service (2021) Basic Forest Statistics 2020. Korea Forest Service, Daejeon, Republic of Korea, 371 pp. https://kfss.forest.go.kr [accessed on 10 December 2025]

[B21] Lee JG, Lee JH, Choi DY, Park SJ, Kim AY, Kim SK (2021a) Four new species of the genus *Pholcus* Walckenaer (Araneae, Pholcidae) from Korea. Journal of Species Research 10(1): 86–98. 10.11646/zootaxa.5052.1.3

[B22] Lee JG, Lee JH, Choi DY, Park SJ, Kim AY, Kim SK (2021b) Five new species of the genus *Pholcus* Walckenaer (Araneae: Pholcidae) from South Korea. Zootaxa 5052(1): 61–77. 10.11646/zootaxa.5052.1.3 34810888

[B23] Lee JG, Lee JH, Choi DY, Park SJ, Baek MJ, Kim SK (2024) Nine new species of the genus *Pholcus* Walckenaer (Araneae: Pholcidae) from South Korea. Zootaxa 5432(2): 179–212. 10.11646/zootaxa.5432.2.3 39645788

[B24] Lee SY, Yoo JS, Jang CM, Kim ST (2025) Five new spider species of the genus *Pholcus* Walckenaer, 1805 (Araneae: Pholcidae) in mountainous mixed forest from Korea. Korean Journal of Environmental Biology 43(3): 242–254. 10.11626/KJEB.2025.43.3.242

[B25] Meng GL, Carvalho LS, Podsiadlowski L, Huber BA (2026) Low coverage whole genome sequencing reveals a new subfamily of daddy long-legs spiders from Brazilian Caatinga (Araneae: Pholcidae). Arthropod Systematics & Phylogeny 84: 95–121. 10.3897/asp.84.e174748

[B26] Namkung J, Kim JP (1990) A new species of the genus *Pholcus* (Araneae: Pholcidae) from Korea. Korean Arachnology 5: 131–137.

[B27] National Geographic Information Institute (2016) The National Atlas of Korea II. Ministry of Land, Infrastructure and Transport: Suwon, Republic of Korea, 243 pp. [ISBN 978-89-93841-22-0] https://www.ngii.go.kr [accessed on 10 August 2025]

[B28] Paik KY (1978a) Araneae. Illustrated Fauna and Flora of Korea. Ministry of Education, Seoul, Republic of Korea, 21, 548 pp.

[B29] Paik KY (1978b) The Pholcidae (Araneae) of Korea. Educational Journal Kyungpook University Korea 20: 113–135.

[B30] Seo BK (2004) A new species of *Pholcus* (Araneae: Pholcidae) from Korea. Korean Journal of Systematic Zoology 20: 73–77.

[B31] Seo BK (2014) Four new species of the genus *Pholcus* (Araneae: Pholcidae) from Korea. Korean Journal of Applied Entomology 53(4): 399–408.

[B32] Seo BK (2018) New species and records of the spider families Pholcidae, Uloboridae, Linyphiidae, Theridiidae, Phrurolithidae, and Thomisidae (Araneae) from Korea. Journal of Species Research 7(4): 251–290. 10.12651/JSR.2018.7.4.251

[B33] Shorthouse DP (2010) SimpleMappr, an Online Tool to Produce Publication-Quality Point Maps. https://www.simplemappr.net [accessed on 8 June 2025]

[B34] World Spider Catalog (2026) World Spider Catalog. Version 26. Natural History Museum Bern. 10.24436/2 [accessed on 5 January 2026]

[B35] Xofis P, Kefalas G, Poirazidis K (2023) Biodiversity and conservation of forests. Forests 14(9): 1871. 10.3390/f14091871

[B36] Yao ZY, Wang X, Li SQ (2021) Tip of the iceberg: species diversity of *Pholcus* spiders (Araneae, Pholcidae) in the Changbai Mountains, northeast China. Zoological Research 42(3): 267–271. [Supplement 60 pp.] 10.24272/j.issn.2095-8137.2021.037 PMC8175958 33797209

[B37] Zhao FY, Jiang T, Yang L, He QQ, Zheng G, Yao ZY (2023) Pholcid spiders of the *Pholcus phungiformes* species-group (Araneae, Pholcidae) from Liaoning Province, China: an overview, with description of a new species. ZooKeys 1156: 1–14. 10.3897/zookeys.1156.98331 PMC10194078 37214272

